# Kirner's deformity of the fifth finger: A case report

**DOI:** 10.1097/MD.0000000000022294

**Published:** 2020-09-25

**Authors:** Ma Tianxiao, Dongyue Wang, Lihua Song

**Affiliations:** Department of Orthopaedic Surgery, The Orthopaedics Hospital of Xingtai City, Xingtai, Hebei, P.R. China.

**Keywords:** child, hand, Kirner's deformity, MRI, osteotomy, radiography, skeletal deformity

## Abstract

**Rationale:**

Kirner's deformity is an uncommon deformity of finger, characterized by palmo-radial curvature of distal phalanx of the fifth finger. The specific mechanism remains unknown yet. This study aims to present a case report to add the knowledge on this type of deformity.

**Patient concerns:**

A 9-year-old girl presenting with deformity of her fifth finger since she was born was admitted to our hand surgery clinic. MRI findings showed widened epiphyseal plate, L-shaped physis, but normal flexor digitorum profundus tendon insertion, without any significantly enhanced soft issues.

**Diagnosis:**

Kirner's deformity of the fifth finger.

**Interventions:**

We presented 2 surgical choices for the patient: one was wedge osteotomy of the distal phalanx to correct the mechanical line of the distal phalanx and fixation with Kirschner wire and the other one was cut-off of deep flexor tendon insertion with brace immobilization, but her guardians refused either of them.

**Outcomes:**

Consecutive follow-up was performed for 19 months after the first visit, showing no any change in finger shape and function.

**Lessons:**

The L-shaped epiphyses may be the cause of Kirner's deformity and further attention should be paid on in the clinic. This case report provided a basis for the etiological diagnosis and future treatment of Kirner's deformity.

## 1 Introduction

Kirner's deformity is a rare deformity of fingers, which was firstly reported by Kirner J in Germany in 1927.^[1]^ It was reported that the incidence rate of Kirner's deformity was extremely low, ranging from 0.15% to 0.25%.^[2,3]^ It is characterized by the palmo-radial curvature of the distal phalanx of the fifth finger.^[4–11]^ Despite the symptom of being painless, the patients may have swelling of distal interphalangeal (DIP) joint and the development of watch-glass nail in the fingers involved.^[5,12,13]^ Among most of the previously reported cases, the deformity usually affected the fifth finger only. However, some case reports also described involvement of other fingers.^[5,6]^ By now, no definite cause has been confirmed for this type of disease. One hypothesis is about abnormal insertion of flexor digitorum profundus tendon,^[3]^ and the other is chronic inflammation and vascularization of soft issues.^[5]^ In another assumption, there is a cartilaginous extension of the physis in Kirner's deformity, which represented a “volar bracketed epiphysis” with an L-shaped physis.^[4]^ In this study, we reported a case of the Kirner's deformity in a 9-year-old girl, and systematically reviewed all the cases reported previously.

## 2 Case presentation

This study was approved by the ethics committee of the Orthopaedics Hospital of Xingtai City, and written informed consent had been obtained from the patient and her guardian, who permitted relevant data published in the journal.

**Figure 1:**
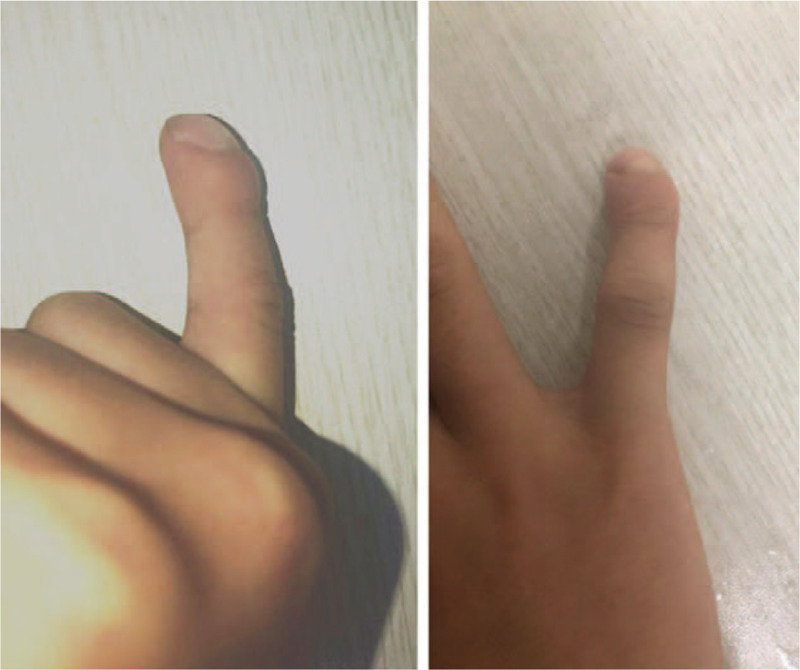
Photograph of the right hand shows volar-radial angular deformity of the distal phalanx of the fifth finger.

**Figure 2:**
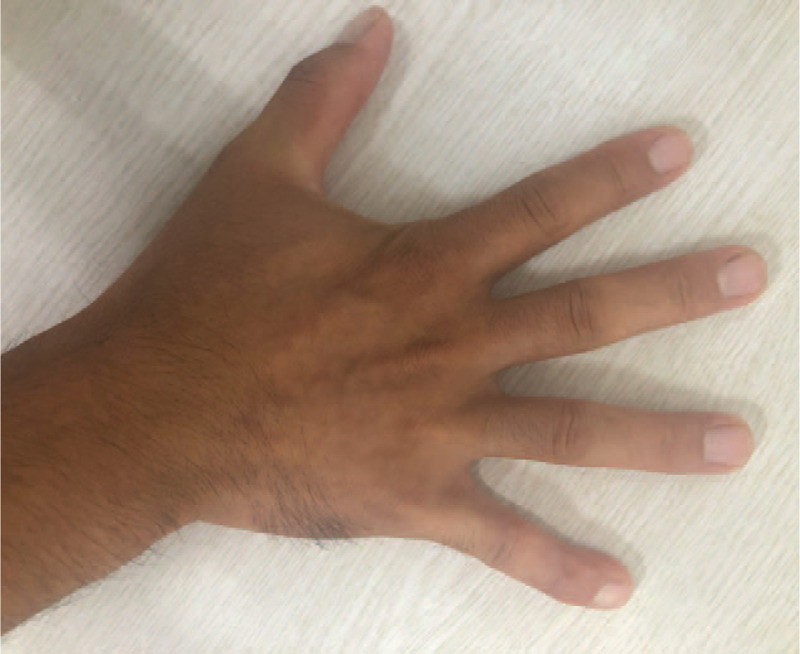
The similar Kirner deformity of the distal fifth finger for the patient’ uncle.

**Figure 3:**
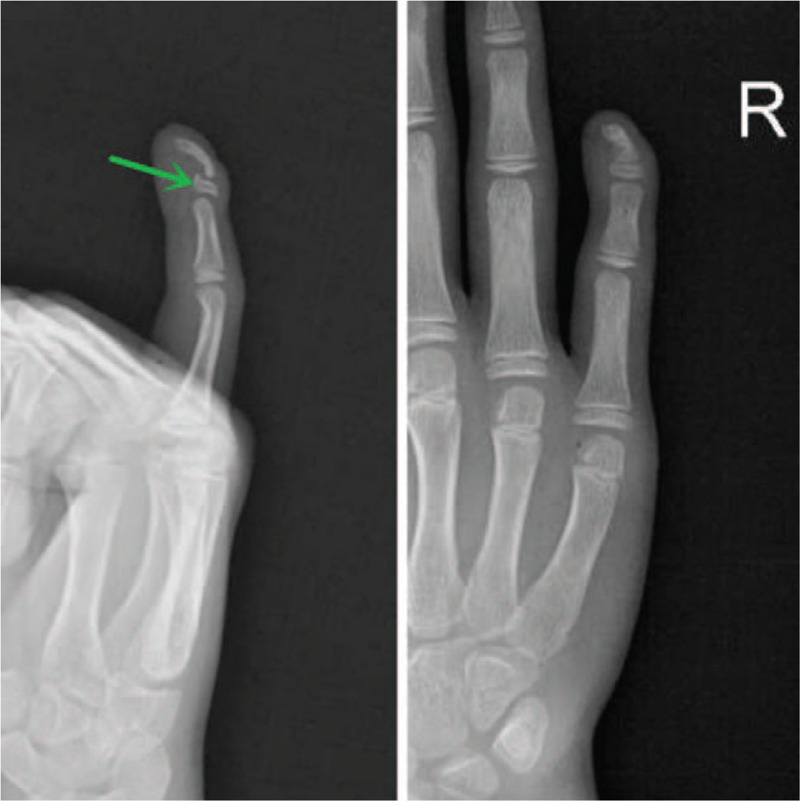
Radiograph shows fifth distal phalanges of the right hand with volar-radial angular deformity, Wider and thicker growth plate of distal phalanx in the form of L-physis (lateral projection).

**Figure 4:**
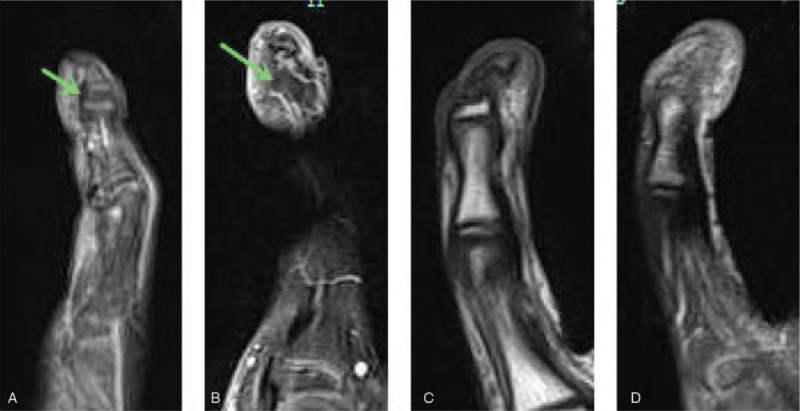
MRI of the right fifth finger, Normal level of tendon insertion (A). Wider and thicker growth plate of distal phalanx in the form of L-physis, but without any enhanced soft tissue (B–D).

A 9-year-old girl was admitted to our hospital with her parents for treatment of little finger deformity of her right hand. She complained about the curvature and deformity of distal phalanx of right fifth finger (Fig. 1). One of her uncles suffered from the same deformity (Fig. 2). The patient had no history of fracture, burn, freezing injury or infectious disease. The clinical manifestations included the palmo-radial curvature of the distal phalanx of right little finger without significant tenderness. Radiograph showed volar-radial angular deformity of distal phalanges of her fifth finger, wider and thicker growth plate of distal phalanx in the form of L-physis (Fig. 3). Laboratory examinations showed no increase in leukocytes, erythrocyte sedimentation rate (ESR), and C-reactive protein, while MRI of fingers revealed slightly widened epiphyseal plate of the distal phalanx of the right little finger, L-shaped physis in epiphysis, and normally inserted flexor digitorum profundus tendon (Fig. 4). No abnormal or enhanced signal was spotted from soft issues even after higher dose was administered.

According to the typical physical examination manifestations and imaging findings, we diagnosed it as Kirner's deformity of the fifth finger. We recommended that patients should be hospitalized for further operative treatment. We provided 2 operation choices, one was wedge osteotomy of the distal phalanx to correct the mechanical line of the distal phalanx and fixation with Kirschner wire, and the other one is cut-off of deep flexor tendon insertion with brace immobilization. However, the patient and her guardians refused our recommendation and were discharged home, because they thought this type of deformity did not affect the daily activities, similar as that her uncle experienced (Fig. 2).

In the subsequent visits, totally lasting 19 months since her first visit, we have not found any change in the finger shape, range of the deformed finger and the ability in daily activities.

## 3 Discussion and conclusions

As an uncommon deformity of finger, Kirner's deformity is characterized by the palmo-radial curvature of the distal phalanx of the fifth finger, and more often seen in women than men.^[4–11]^ It may be present by heredity as autosomal dominant trait with incomplete penetrance. The homozygous state determines the manifestation of deformity of more than one finger and even in both hands. Based on this, Song et al and Koh et al proposed 2 ways of classification for this disease. The first one is regarding congenital anomaly that is mostly inherited in the family, which usually lasts for several months or years and then the deformity disappears after closure of epiphysis without affecting the function of the finger. The other is acquired, which is not found in other members of the family and usually attacks the patient for the first time at his or her 10 years old. Nevertheless, some researchers also found more complicated interplay between the hereditary susceptibility and clinical manifestations.^[7,14]^

Kirner's disease should be distinguished from other similar diseases, including camptodactyly, deformity of flexion at the PIP joint, and clinodactyly, radial deviation at the DIP joint. Literature review revealed that this deformity might be associated with Turner's syndrome, Cornelia de Lange syndrome, Down syndrome and Silver syndrome.^[12,15–17]^

The pathogenesis of Kirner's deformity remains poorly understood. One of the assumptions is that Kirner's deformity may result from an unusually distal insertion of flexor digitorum profundus tendon along the palmar surface of nearly the entire distal phalanx. The flexor tendon may exert forces acting on the growing bone in the volar and radial direction, resulting in a flexion deformity prior to fusion of the physis.^[3]^ On the theoretical basis above, Benatar et al treated the disease by removing the stops of flexor digitorum profundus tendon, and reported anatomic variations during surgery as well as promising postoperative clinical efficacy.^[3]^ On the contrary, our case hereby is not consistent with the case described by Benatar et al, but similar as that depicted by Lee^[7]^ where MRI revealed normal insertion site of flexor digitorum profundus tendon. Given the deduced deviation force of flexor digitorum profundus tendon from epiphysis, damage to the epiphysis of distal phalanx of the fifth finger during tendon detachment maybe the primary reason for improvement of affected finger after the surgery.^[4,7,13]^ As a result, the postsurgical performance cannot shed any light on the pathogenesis of Kirner's deformity.

Another assumption is bending of joints, and deformity of distal phalanx seems to result from a chronic inflammatory process that starts in the soft tissues of distal fingers.^[5]^ Miller^[11]^ pointed out in 2004 that there were 2 key problems with this assumption. Firstly, the diagnosis was wrong, because the Kirner's deformity could involve several fingers of both hands, as reported by Brune et al in 2003. Therefore, it is more appropriate that it should be diagnosed as Camptodactyly instead. Secondly, the “substantial” soft tissue enhancement as shown on the T1-W FSGad images may represent a commonly seen artifact when performing fat-saturation operation on the small curved area. By placing saline bags along the small parts or curved surfaces so as to provide increased magnetic field uniformity and remove the artifact, we got an outcome that was completely different from that of Brune et al. Our MRI indicated wider and thicker growth plate of distal phalanx in the form of L-physis, but without any enhanced soft tissue (Fig. 3).

According to the third assumption for Kirner's deformity, there is a cartilaginous extension representing a “volar bracketed epiphysis” with an L-shaped physis.^[4,13]^ This theory highlighted that cartilaginous extension of the physis indicated a “volar bracketed epiphysis” with an L-shaped rather than C-shaped physis. The morphological difference may be attributed to following several conditions. In the first place, as for clinodactyly, the bracket joins with the distal articular cartilage to complete the “C”, whereas as for Kirner's deformity the “C” is incomplete because the tip of the distal phalanx lacks articular cartilage, therefore developing as a result of intra-membranous rather than intra-cartilaginous ossification. Then, the L-shaped physis of Kirner's deformity appears to be limited distally by the FDP insertion. We believe this L-shaped physis may act as an anlage, restricting growth of the proximal volar metaphysis; and together with continued dorsal growth finally resulted in volar bowing of the distal phalanx. After reviewing all the previous reports concerning Kirner's deformity, the physical checking results, and the combined MRI and X-ray findings of our case, we believe this assumption is convincible (Figs. 2 and 3).

The existing treatment choices for Kirner's deformity included early fixation with delayed continuous splinting before maturity,^[3,14,18]^ removal of stops of flexor digitorum profundus tendon,^[3]^ corrective osteotomy,^[4]^ and excision of excessive epiphysis along palm to achieve bony alignment.^[4,13]^ As to early fixation with delayed continuous splinting before maturity, strict guardianship and follow-up are required for realizing effective prevention and correction of the deformity. For removal of stops of flexor digitorum profundus tendon, we think it sets to correct the deformity by reducing the deviation of flexor digitorum profundus tendon from epiphysis and damaging the epiphysis of distal phalanx. However, the natural retraction of flexor digitorum profundus tendon may cause a permanent loss of DIP joint. Corrective osteotomy has to be performed until the epiphysis becomes completely closed; otherwise, a relapse may occur. Furthermore, the primary goal of the surgery is to improve the morphology instead of function of the involved finger. As for excision of palmar excessive epiphysis, there remains lack of clinical data to support, though it is deemed as feasible.

Taken together, the pathogenesis of Kirner's deformity remains unclear. To our knowledge, we believe there is a cartilaginous extension of physis in this condition, which represents a “volar bracketed epiphysis”, with an L-shaped physis. We suggest the volar splint fixation transversely or resection procedure for treatment of such deformity to achieve bony alignment. Long-term follow-up results of this patients are warranted, despite she did not receive surgical treatment.

## Acknowledgments

We are grateful to YQH and QBB of the Department of Orthopedics, and to QW and JBY of the Department of medical imaging for their kind assistance. All the authors declare that they have no conflict of interest.

## Author contributions

LHS designed the study; LHS and TXM analysed the Imaging data; DYW and TXM seared the relevant literature. TXM wrote the manuscript and LHS approved the manuscript.
